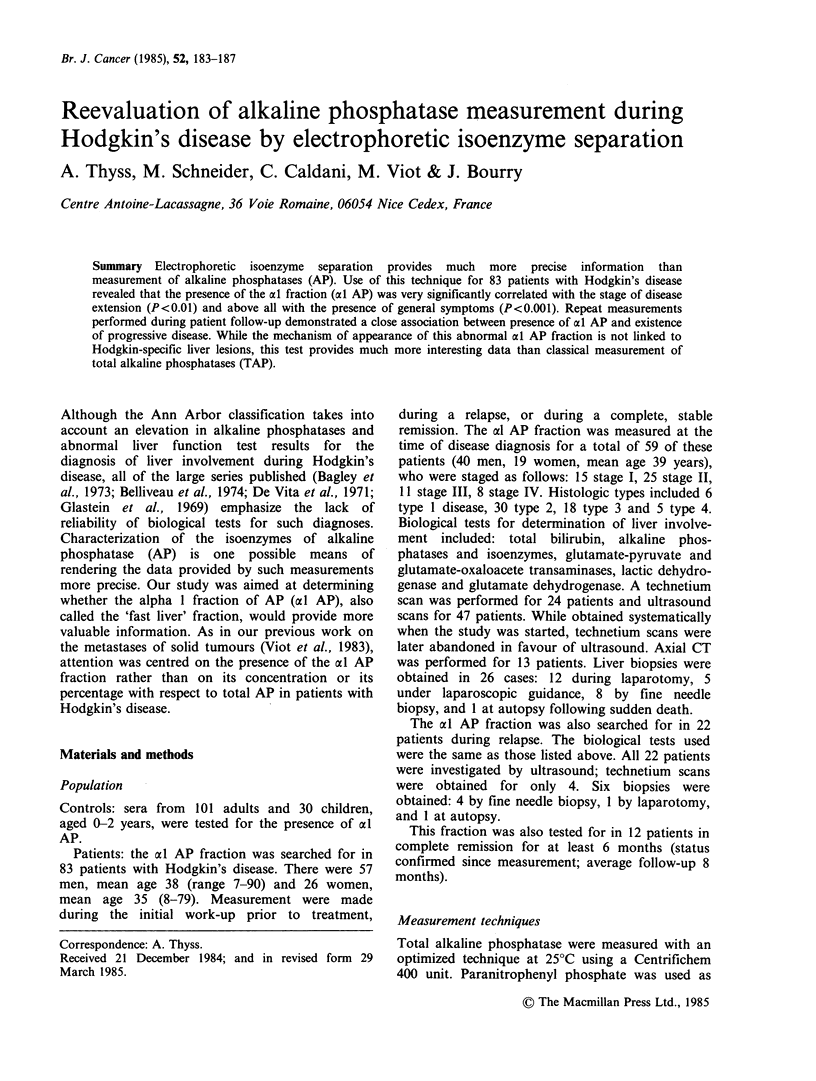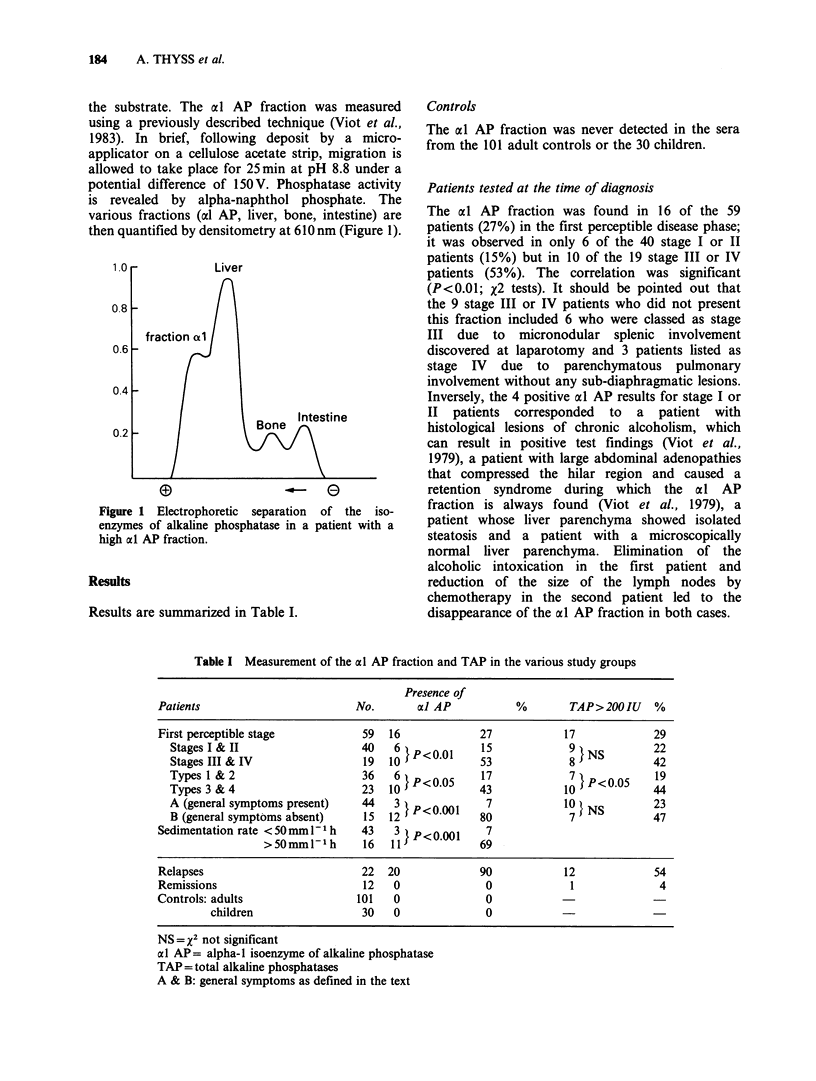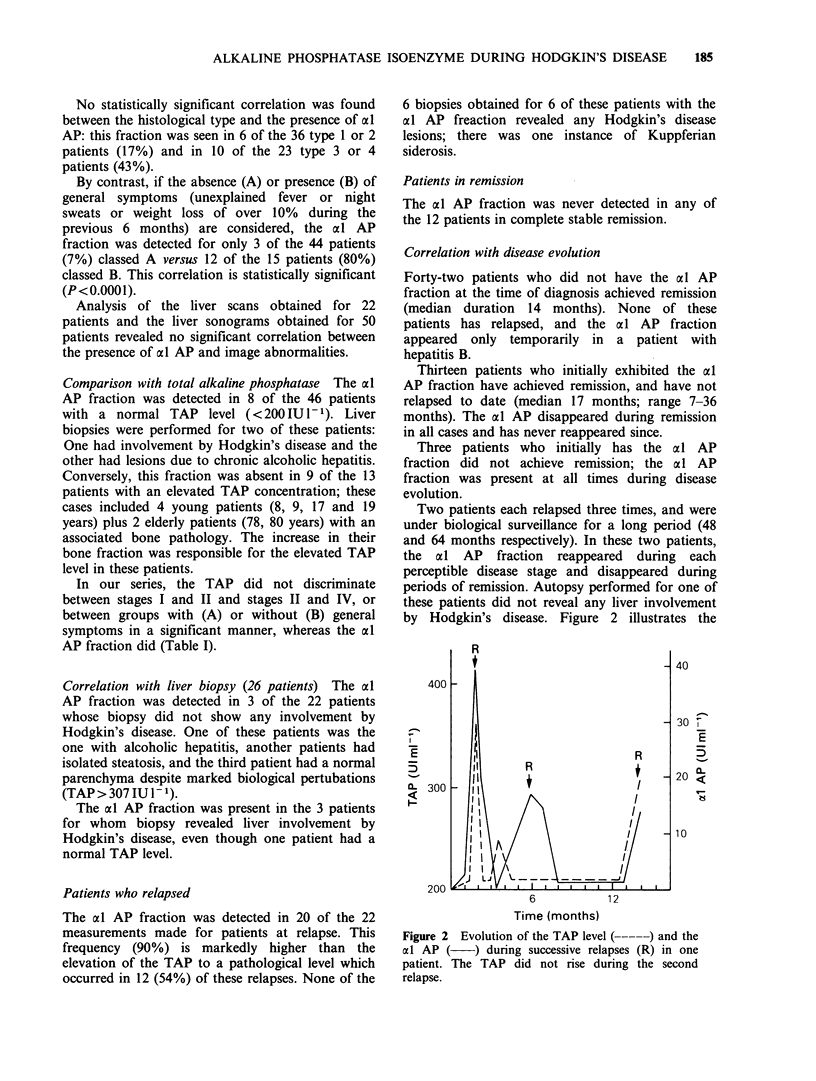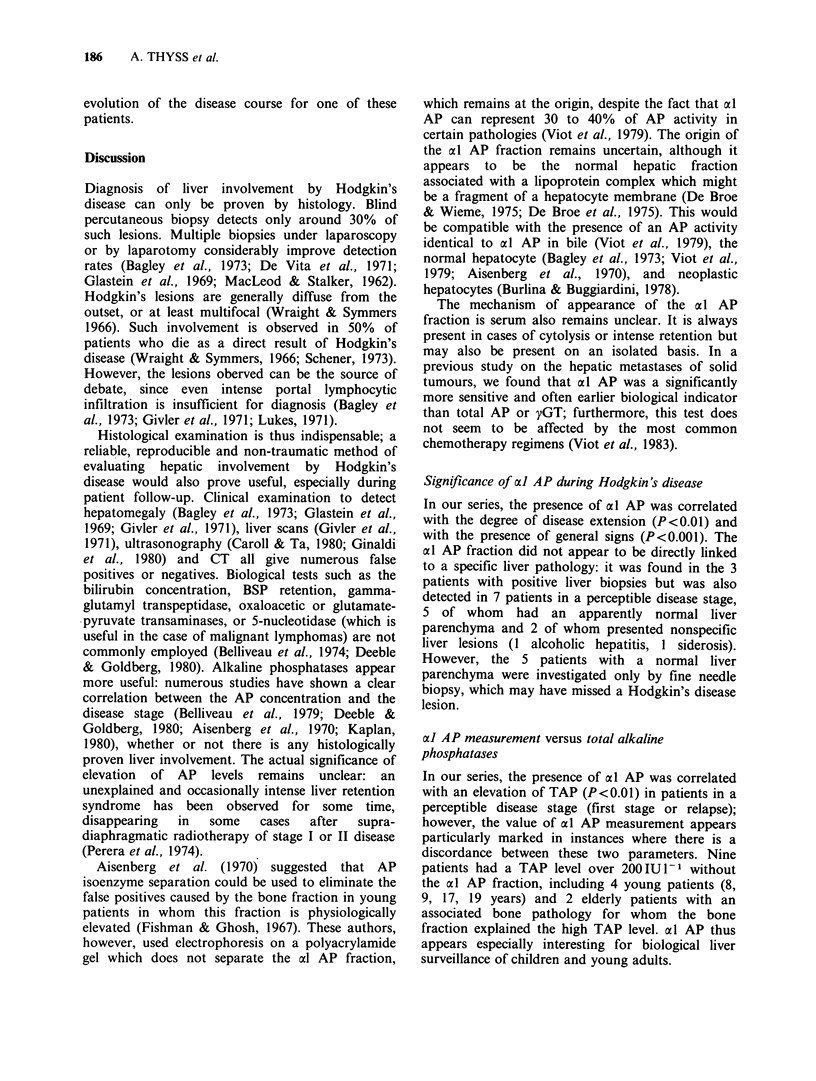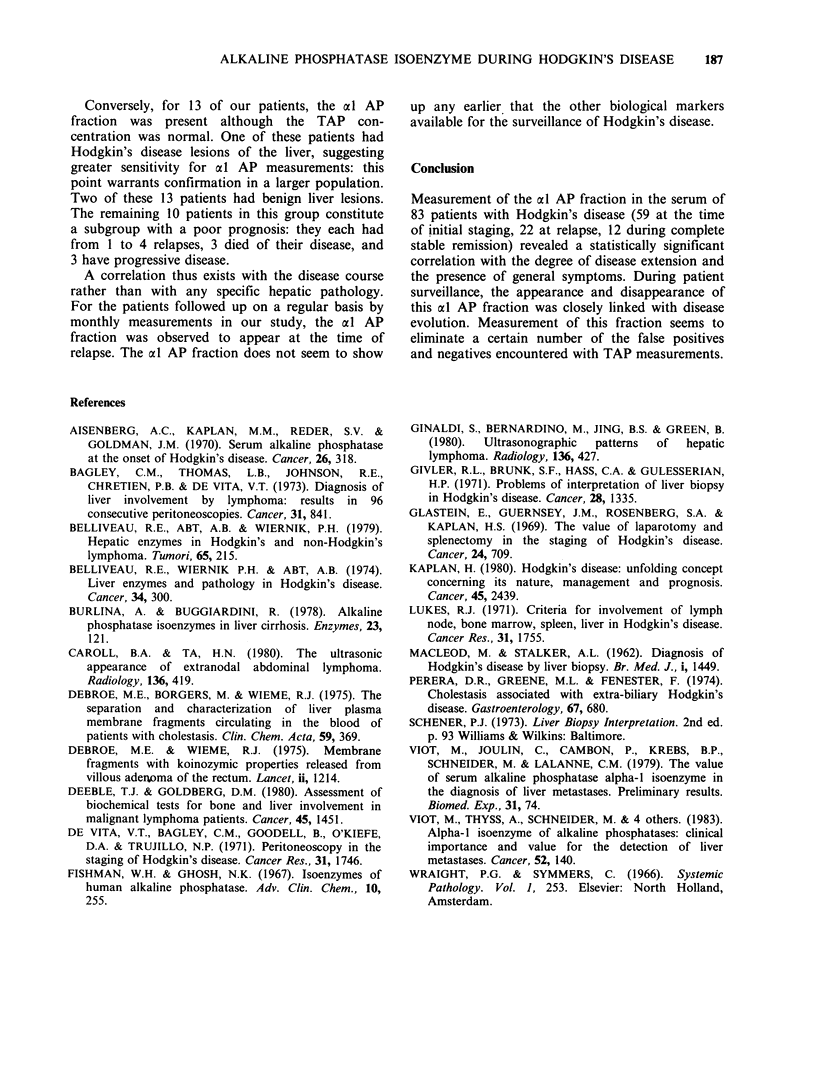# Reevaluation of alkaline phosphatase measurement during Hodgkin's disease by electrophoretic isoenzyme separation.

**DOI:** 10.1038/bjc.1985.176

**Published:** 1985-08

**Authors:** A. Thyss, M. Schneider, C. Caldani, M. Viot, J. Bourry

## Abstract

Electrophoretic isoenzyme separation provides much more precise information than measurement of alkaline phosphatases (AP). Use of this technique for 83 patients with Hodgkin's disease revealed that the presence of the alpha 1 fraction (alpha 1 AP) was very significantly correlated with the stage of disease extension (P less than 0.01) and above all with the presence of general symptoms (P less than 0.001). Repeat measurements performed during patient follow-up demonstrated a close association between presence of alpha 1 AP and existence of progressive disease. While the mechanism of appearance of this abnormal alpha 1 AP fraction is not linked to Hodgkin-specific liver lesions, this test provides much more interesting data than classical measurement of total alkaline phosphatases (TAP).


*Br. J. Cancer* (1985), **52**, 183–187

### Reevaluation of alkaline phosphatase measurement during Hodgkin's disease by electrophoretic isoenzyme separation

A. Thyss, M. Schneider, C. Caldani, M. Viot & J. Bourry

*Centre Antoine-Lacassagne, 36 Voie Romaine, 06054 Nice Cedex, France*

**Summary** Electrophoretic isoenzyme separation provides much more precise information than measurement of alkaline phosphatases (AP). Use of this technique for 83 patients with Hodgkin's disease revealed that the presence of the α1 fraction (α1 AP) was very significantly correlated with the stage of disease extension ($P<0.01$) and above all with the presence of general symptoms ($P<0.001$). Repeat measurements performed during patient follow-up demonstrated a close association between presence of α1 AP and existence of progressive disease. While the mechanism of appearance of this abnormal α1 AP fraction is not linked to Hodgkin-specific liver lesions, this test provides much more interesting data than classical measurement of total alkaline phosphatases (TAP).

Although the Ann Arbor classification takes into account an elevation in alkaline phosphatases and abnormal liver function test results for the diagnosis of liver involvement during Hodgkin's disease, all of the large series published (Bagley *et al.*, 1973; Belliveau *et al.*, 1974; De Vita *et al.*, 1971; Glastein *et al.*, 1969) emphasize the lack of reliability of biological tests for such diagnoses. Characterization of the isoenzymes of alkaline phosphatase (AP) is one possible means of rendering the data provided by such measurements more precise. Our study was aimed at determining whether the alpha 1 fraction of AP (α1 AP), also called the 'fast liver' fraction, would provide more valuable information. As in our previous work on the metastases of solid tumours (Viot *et al.*, 1983), attention was centred on the presence of the α1 AP fraction rather than on its concentration or its percentage with respect to total AP in patients with Hodgkin's disease.

### Materials and methods

### *Population*

Controls: sera from 101 adults and 30 children, aged 0–2 years, were tested for the presence of α1 AP.

Patients: the α1 AP fraction was searched for in 83 patients with Hodgkin's disease. There were 57 men, mean age 38 (range 7–90) and 26 women, mean age 35 (8–79). Measurement were made during the initial work-up prior to treatment, during a relapse, or during a complete, stable remission. The α1 AP fraction was measured at the time of disease diagnosis for a total of 59 of these patients (40 men, 19 women, mean age 39 years), who were staged as follows: 15 stage I, 25 stage II, 11 stage III, 8 stage IV. Histologic types included 6 type 1 disease, 30 type 2, 18 type 3 and 5 type 4. Biological tests for determination of liver involvement included: total bilirubin, alkaline phosphatases and isoenzymes, glutamate-pyruvate and glutamate-oxaloacete transaminases, lactic dehydrogenase and glutamate dehydrogenase. A technetium scan was performed for 24 patients and ultrasound scans for 47 patients. While obtained systematically when the study was started, technetium scans were later abandoned in favour of ultrasound. Axial CT was performed for 13 patients. Liver biopsies were obtained in 26 cases: 12 during laparotomy, 5 under laparoscopic guidance, 8 by fine needle biopsy, and 1 at autopsy following sudden death.

The α1 AP fraction was also searched for in 22 patients during relapse. The biological tests used were the same as those listed above. All 22 patients were investigated by ultrasound; technetium scans were obtained for only 4. Six biopsies were obtained: 4 by fine needle biopsy, 1 by laparotomy, and 1 at autopsy.

This fraction was also tested for in 12 patients in complete remission for at least 6 months (status confirmed since measurement; average follow-up 8 months).

### *Measurement techniques*

Total alkaline phosphatase were measured with an optimized technique at 25°C using a Centrifichem 400 unit. Paranitrophenyl phosphate was used as

Correspondence: A. Thyss.
Received 21 December 1984; and in revised form 29 March 1985.

© The Macmillan Press Ltd., 1985

the substrate. The α1 AP fraction was measured using a previously described technique (Viot *et al.*, 1983). In brief, following deposit by a micro-applicator on a cellulose acetate strip, migration is allowed to take place for 25 min at pH 8.8 under a potential difference of 150 V. Phosphatase activity is revealed by alpha-naphthol phosphate. The various fractions (α1 AP, liver, bone, intestine) are then quantified by densitometry at 610 nm (Figure 1).

**Figure 1** Electrophoretic separation of the iso-enzymes of alkaline phosphatase in a patient with a high α1 AP fraction.

### Results

Results are summarized in Table I.

### *Controls*

The α1 AP fraction was never detected in the sera from the 101 adult controls or the 30 children.

### *Patients tested at the time of diagnosis*

The α1 AP fraction was found in 16 of the 59 patients (27%) in the first perceptible disease phase; it was observed in only 6 of the 40 stage I or II patients (15%) but in 10 of the 19 stage III or IV patients (53%). The correlation was significant ($P<0.01$; $\chi2$ tests). It should be pointed out that the 9 stage III or IV patients who did not present this fraction included 6 who were classed as stage III due to micronodular splenic involvement discovered at laparotomy and 3 patients listed as stage IV due to parenchymatous pulmonary involvement without any sub-diaphragmatic lesions. Inversely, the 4 positive α1 AP results for stage I or II patients corresponded to a patient with histological lesions of chronic alcoholism, which can result in positive test findings (Viot *et al.*, 1979), a patient with large abdominal adenopathies that compressed the hilar region and caused a retention syndrome during which the α1 AP fraction is always found (Viot *et al.*, 1979), a patient whose liver parenchyma showed isolated steatosis and a patient with a microscopically normal liver parenchyma. Elimination of the alcoholic intoxication in the first patient and reduction of the size of the lymph nodes by chemotherapy in the second patient led to the disappearance of the α1 AP fraction in both cases.

**Table I** Measurement of the α1 AP fraction and TAP in the various study groups

| *Patients* | *No.* | *Presence of α1 AP* | | % | *TAP>200 IU* | | % |
|---|---|---|---|---|---|---|---|
| First perceptible stage | 59 | 16 | | 27 | 17 | | 29 |
| Stages I & II | 40 | 6 | $P<0.01$ | 15 | 9 | NS | 22 |
| Stages III & IV | 19 | 10 | | 53 | 8 | | 42 |
| Types 1 & 2 | 36 | 6 | $P<0.05$ | 17 | 7 | $P<0.05$ | 19 |
| Types 3 & 4 | 23 | 10 | | 43 | 10 | | 44 |
| A (general symptoms present) | 44 | 3 | $P<0.001$ | 7 | 10 | NS | 23 |
| B (general symptoms absent) | 15 | 12 | | 80 | 7 | | 47 |
| Sedimentation rate $<50\,mm\,l^{-1}\,h$ | 43 | 3 | $P<0.001$ | 7 | | | |
| $>50\,mm\,l^{-1}\,h$ | 16 | 11 | | 69 | | | |
| Relapses | 22 | 20 | | 90 | 12 | | 54 |
| Remissions | 12 | 0 | | 0 | 1 | | 4 |
| Controls: adults | 101 | 0 | | 0 | — | | — |
| children | 30 | 0 | | 0 | — | | — |

NS = $\chi^2$ not significant
α1 AP = alpha-1 isoenzyme of alkaline phosphatase
TAP = total alkaline phosphatases
A & B: general symptoms as defined in the text

No statistically significant correlation was found between the histological type and the presence of α1 AP: this fraction was seen in 6 of the 36 type 1 or 2 patients (17%) and in 10 of the 23 type 3 or 4 patients (43%).

By contrast, if the absence (A) or presence (B) of general symptoms (unexplained fever or night sweats or weight loss of over 10% during the previous 6 months) are considered, the α1 AP fraction was detected for only 3 of the 44 patients (7%) classed A *versus* 12 of the 15 patients (80%) classed B. This correlation is statistically significant ($P<0.0001$).

Analysis of the liver scans obtained for 22 patients and the liver sonograms obtained for 50 patients revealed no significant correlation between the presence of α1 AP and image abnormalities.

*Comparison with total alkaline phosphatase* The α1 AP fraction was detected in 8 of the 46 patients with a normal TAP level ($<200\,IU\,l^{-1}$). Liver biopsies were performed for two of these patients: One had involvement by Hodgkin's disease and the other had lesions due to chronic alcoholic hepatitis. Conversely, this fraction was absent in 9 of the 13 patients with an elevated TAP concentration; these cases included 4 young patients (8, 9, 17 and 19 years) plus 2 elderly patients (78, 80 years) with an associated bone pathology. The increase in their bone fraction was responsible for the elevated TAP level in these patients.

In our series, the TAP did not discriminate between stages I and II and stages II and IV, or between groups with (A) or without (B) general symptoms in a significant manner, whereas the α1 AP fraction did (Table I).

*Correlation with liver biopsy (26 patients)* The α1 AP fraction was detected in 3 of the 22 patients whose biopsy did not show any involvement by Hodgkin's disease. One of these patients was the one with alcoholic hepatitis, another patients had isolated steatosis, and the third patient had a normal parenchyma despite marked biological pertubations ($TAP>307\,IU\,l^{-1}$).

The α1 AP fraction was present in the 3 patients for whom biopsy revealed liver involvement by Hodgkin's disease, even though one patient had a normal TAP level.

*Patients who relapsed*

The α1 AP fraction was detected in 20 of the 22 measurements made for patients at relapse. This frequency (90%) is markedly higher than the elevation of the TAP to a pathological level which occurred in 12 (54%) of these relapses. None of the 6 biopsies obtained for 6 of these patients with the α1 AP freaction revealed any Hodgkin's disease lesions; there was one instance of Kuppferian siderosis.

*Patients in remission*

The α1 AP fraction was never detected in any of the 12 patients in complete stable remission.

*Correlation with disease evolution*

Forty-two patients who did not have the α1 AP fraction at the time of diagnosis achieved remission (median duration 14 months). None of these patients has relapsed, and the α1 AP fraction appeared only temporarily in a patient with hepatitis B.

Thirteen patients who initially exhibited the α1 AP fraction have achieved remission, and have not relapsed to date (median 17 months; range 7–36 months). The α1 AP disappeared during remission in all cases and has never reappeared since.

Three patients who initially has the α1 AP fraction did not achieve remission; the α1 AP fraction was present at all times during disease evolution.

Two patients each relapsed three times, and were under biological surveillance for a long period (48 and 64 months respectively). In these two patients, the α1 AP fraction reappeared during each perceptible disease stage and disappeared during periods of remission. Autopsy performed for one of these patients did not reveal any liver involvement by Hodgkin's disease. Figure 2 illustrates the

**Figure 2** Evolution of the TAP level (-----) and the α1 AP (——) during successive relapses (R) in one patient. The TAP did not rise during the second relapse.

evolution of the disease course for one of these patients.

### Discussion

Diagnosis of liver involvement by Hodgkin's disease can only be proven by histology. Blind percutaneous biopsy detects only around 30% of such lesions. Multiple biopsies under laparoscopy or by laparotomy considerably improve detection rates (Bagley *et al.*, 1973; De Vita *et al.*, 1971; Glastein *et al.*, 1969; MacLeod & Stalker, 1962). Hodgkin's lesions are generally diffuse from the outset, or at least multifocal (Wraight & Symmers 1966). Such involvement is observed in 50% of patients who die as a direct result of Hodgkin's disease (Wraight & Symmers, 1966; Schener, 1973). However, the lesions observed can be the source of debate, since even intense portal lymphocytic infiltration is insufficient for diagnosis (Bagley *et al.*, 1973; Givler *et al.*, 1971; Lukes, 1971).

Histological examination is thus indispensable; a reliable, reproducible and non-traumatic method of evaluating hepatic involvement by Hodgkin's disease would also prove useful, especially during patient follow-up. Clinical examination to detect hepatomegaly (Bagley *et al.*, 1973; Glastein *et al.*, 1969; Givler *et al.*, 1971), liver scans (Givler *et al.*, 1971), ultrasonography (Caroll & Ta, 1980; Ginaldi *et al.*, 1980) and CT all give numerous false positives or negatives. Biological tests such as the bilirubin concentration, BSP retention, gamma-glutamyl transpeptidase, oxaloacetic or glutamate-pyruvate transaminases, or 5-nucleotidase (which is useful in the case of malignant lymphomas) are not commonly employed (Belliveau *et al.*, 1974; Deeble & Goldberg, 1980). Alkaline phosphatases appear more useful: numerous studies have shown a clear correlation between the AP concentration and the disease stage (Belliveau *et al.*, 1979; Deeble & Goldberg, 1980; Aisenberg *et al.*, 1970; Kaplan, 1980), whether or not there is any histologically proven liver involvement. The actual significance of elevation of AP levels remains unclear: an unexplained and occasionally intense liver retention syndrome has been observed for some time, disappearing in some cases after supra-diaphragmatic radiotherapy of stage I or II disease (Perera *et al.*, 1974).

Aisenberg *et al.* (1970) suggested that AP isoenzyme separation could be used to eliminate the false positives caused by the bone fraction in young patients in whom this fraction is physiologically elevated (Fishman & Ghosh, 1967). These authors, however, used electrophoresis on a polyacrylamide gel which does not separate the $\alpha 1$ AP fraction, which remains at the origin, despite the fact that $\alpha 1$ AP can represent 30 to 40% of AP activity in certain pathologies (Viot *et al.*, 1979). The origin of the $\alpha 1$ AP fraction remains uncertain, although it appears to be the normal hepatic fraction associated with a lipoprotein complex which might be a fragment of a hepatocyte membrane (De Broe & Wieme, 1975; De Broe *et al.*, 1975). This would be compatible with the presence of an AP activity identical to $\alpha 1$ AP in bile (Viot *et al.*, 1979), the normal hepatocyte (Bagley *et al.*, 1973; Viot *et al.*, 1979; Aisenberg *et al.*, 1970), and neoplastic hepatocytes (Burlina & Buggiardini, 1978).

The mechanism of appearance of the $\alpha 1$ AP fraction is serum also remains unclear. It is always present in cases of cytolysis or intense retention but may also be present on an isolated basis. In a previous study on the hepatic metastases of solid tumours, we found that $\alpha 1$ AP was a significantly more sensitive and often earlier biological indicator than total AP or $\gamma$GT; furthermore, this test does not seem to be affected by the most common chemotherapy regimens (Viot *et al.*, 1983).

### *Significance of $\alpha 1$ AP during Hodgkin's disease*

In our series, the presence of $\alpha 1$ AP was correlated with the degree of disease extension ($P<0.01$) and with the presence of general signs ($P<0.001$). The $\alpha 1$ AP fraction did not appear to be directly linked to a specific liver pathology: it was found in the 3 patients with positive liver biopsies but was also detected in 7 patients in a perceptible disease stage, 5 of whom had an apparently normal liver parenchyma and 2 of whom presented nonspecific liver lesions (1 alcoholic hepatitis, 1 siderosis). However, the 5 patients with a normal liver parenchyma were investigated only by fine needle biopsy, which may have missed a Hodgkin's disease lesion.

### *$\alpha 1$ AP measurement* versus *total alkaline phosphatases*

In our series, the presence of $\alpha 1$ AP was correlated with an elevation of TAP ($P<0.01$) in patients in a perceptible disease stage (first stage or relapse); however, the value of $\alpha 1$ AP measurement appears particularly marked in instances where there is a discordance between these two parameters. Nine patients had a TAP level over $200 \, IU \, l^{-1}$ without the $\alpha 1$ AP fraction, including 4 young patients (8, 9, 17, 19 years) and 2 elderly patients with an associated bone pathology for whom the bone fraction explained the high TAP level. $\alpha 1$ AP thus appears especially interesting for biological liver surveillance of children and young adults.

Conversely, for 13 of our patients, the $\alpha 1$ AP fraction was present although the TAP concentration was normal. One of these patients had Hodgkin's disease lesions of the liver, suggesting greater sensitivity for $\alpha 1$ AP measurements: this point warrants confirmation in a larger population. Two of these 13 patients had benign liver lesions. The remaining 10 patients in this group constitute a subgroup with a poor prognosis: they each had from 1 to 4 relapses, 3 died of their disease, and 3 have progressive disease.

A correlation thus exists with the disease course rather than with any specific hepatic pathology. For the patients followed up on a regular basis by monthly measurements in our study, the $\alpha 1$ AP fraction was observed to appear at the time of relapse. The $\alpha 1$ AP fraction does not seem to show up any earlier that the other biological markers available for the surveillance of Hodgkin's disease.

### Conclusion

Measurement of the $\alpha 1$ AP fraction in the serum of 83 patients with Hodgkin's disease (59 at the time of initial staging, 22 at relapse, 12 during complete stable remission) revealed a statistically significant correlation with the degree of disease extension and the presence of general symptoms. During patient surveillance, the appearance and disappearance of this $\alpha 1$ AP fraction was closely linked with disease evolution. Measurement of this fraction seems to eliminate a certain number of the false positives and negatives encountered with TAP measurements.

### References

AISENBERG, A.C., KAPLAN, M.M., REDER, S.V. & GOLDMAN, J.M. (1970). Serum alkaline phosphatase at the onset of Hodgkin's disease. *Cancer*, **26**, 318.

BAGLEY, C.M., THOMAS, L.B., JOHNSON, R.E., CHRETIEN, P.B. & DE VITA, V.T. (1973). Diagnosis of liver involvement by lymphoma: results in 96 consecutive peritoneoscopies. *Cancer*, **31**, 841.

BELLIVEAU, R.E., ABT, A.B. & WIERNIK, P.H. (1979). Hepatic enzymes in Hodgkin's and non-Hodgkin's lymphoma. *Tumori*, **65**, 215.

BELLIVEAU, R.E., WIERNIK P.H. & ABT, A.B. (1974). Liver enzymes and pathology in Hodgkin's disease. *Cancer*, **34**, 300.

BURLINA, A. & BUGGIARDINI, R. (1978). Alkaline phosphatase isoenzymes in liver cirrhosis. *Enzymes*, **23**, 121.

CAROLL, B.A. & TA, H.N. (1980). The ultrasonic appearance of extranodal abdominal lymphoma. *Radiology*, **136**, 419.

DEBROE, M.E., BORGERS, M. & WIEME, R.J. (1975). The separation and characterization of liver plasma membrane fragments circulating in the blood of patients with cholestasis. *Clin. Chem. Acta*, **59**, 369.

DEBROE, M.E. & WIEME, R.J. (1975). Membrane fragments with koinozymic properties released from villous adenoma of the rectum. *Lancet*, **ii**, 1214.

DEEBLE, T.J. & GOLDBERG, D.M. (1980). Assessment of biochemical tests for bone and liver involvement in malignant lymphoma patients. *Cancer*, **45**, 1451.

DE VITA, V.T., BAGLEY, C.M., GOODELL, B., O'KIEFE, D.A. & TRUJILLO, N.P. (1971). Peritoneoscopy in the staging of Hodgkin's disease. *Cancer Res.*, **31**, 1746.

FISHMAN, W.H. & GHOSH, N.K. (1967). Isoenzymes of human alkaline phosphatase. *Adv. Clin. Chem.*, **10**, 255.

GINALDI, S., BERNARDINO, M., JING, B.S. & GREEN, B. (1980). Ultrasonographic patterns of hepatic lymphoma. *Radiology*, **136**, 427.

GIVLER, R.L., BRUNK, S.F., HASS, C.A. & GULESSERIAN, H.P. (1971). Problems of interpretation of liver biopsy in Hodgkin's disease. *Cancer*, **28**, 1335.

GLASTEIN, E., GUERNSEY, J.M., ROSENBERG, S.A. & KAPLAN, H.S. (1969). The value of laparotomy and splenectomy in the staging of Hodgkin's disease. *Cancer*, **24**, 709.

KAPLAN, H. (1980). Hodgkin's disease: unfolding concept concerning its nature, management and prognosis. *Cancer*, **45**, 2439.

LUKES, R.J. (1971). Criteria for involvement of lymph node, bone marrow, spleen, liver in Hodgkin's disease. *Cancer Res.*, **31**, 1755.

MACLEOD, M. & STALKER, A.L. (1962). Diagnosis of Hodgkin's disease by liver biopsy. *Br. Med. J.*, **i**, 1449.

PERERA, D.R., GREENE, M.L. & FENESTER, F. (1974). Cholestasis associated with extra-biliary Hodgkin's disease. *Gastroenterology*, **67**, 680.

SCHENER, P.J. (1973). *Liver Biopsy Interpretation*. 2nd ed. p. 93 Williams & Wilkins: Baltimore.

VIOT, M., JOULIN, C., CAMBON, P., KREBS, B.P., SCHNEIDER, M. & LALANNE, C.M. (1979). The value of serum alkaline phosphatase alpha-1 isoenzyme in the diagnosis of liver metastases. Preliminary results. *Biomed. Exp.*, **31**, 74.

VIOT, M., THYSS, A., SCHNEIDER, M. & 4 others. (1983). Alpha-1 isoenzyme of alkaline phosphatases: clinical importance and value for the detection of liver metastases. *Cancer*, **52**, 140.

WRAIGHT, P.G. & SYMMERS, C. (1966). *Systemic Pathology. Vol. 1*, 253. Elsevier: North Holland, Amsterdam.